# Flavokawain A is a natural inhibitor of PRMT5 in bladder cancer

**DOI:** 10.1186/s13046-022-02500-4

**Published:** 2022-10-05

**Authors:** Shuangjie Liu, Zhuonan Liu, Chiyuan Piao, Zhe Zhang, Chuize Kong, Lei Yin, Xi Liu

**Affiliations:** Department of Urology, The First Hospital of China Medical University, Shenyang, 110001 Liaoning China

**Keywords:** Protein arginine methyltransferase, Flavokawain A, Bladder cancer, Methylation disorder, Arginine methylation

## Abstract

**Background:**

Protein arginine methyltransferases (PRMTs) regulate protein biological activity by modulating arginine methylation in cancer and are increasingly recognized as potential drug targets. Inhibitors targeting PRMTs are currently in the early phases of clinical trials and more candidate drugs are needed. Flavokawain A (FKA), extracted from kava plant, has been recognized as a potential chemotherapy drug in bladder cancer (BC), but its action mechanism remains unclear.

**Methods:**

We first determined the role of a type II PRMT, PRMT5, in BC tissue samples and performed cytological experiments. We then utilized bioinformatics tools, including computational simulation, virtual screening, molecular docking, and energy analysis, to identify the potential use of PRMT5 inhibitors for BC treatment. In vitro and in vivo co-IP and mutation assays were performed to elucidate the molecular mechanism of PRMT5 inhibitor. Pharmacology experiments like bio-layer interferometry, CETSA, and pull-down assays were further used to provide direct evidence of the complex binding process.

**Results:**

Among PRMTs, PRMT5 was identified as a therapeutic target for BC. PRMT5 expression in BC was correlated with poor prognosis and manipulating its expression could affect cancer cell growth. Through screening and extensive experimental validation, we recognized that a natural product, FKA, was a small new inhibitor molecule for PRMT5. We noticed that the product could inhibit the action of BC, in vitro and in vivo, by inhibiting PRMT5. We further demonstrated that FKA blocks the symmetric arginine dimethylation of histone H2A and H4 by binding to Y304 and F580 of PRMT5.

**Conclusions:**

In summary, our research strongly suggests that PRMT5 is a potential epigenetic therapeutic target in bladder cancer, and that FKA can be used as a targeted inhibitor of PRMT5 for the treatment of bladder cancer.

**Supplementary Information:**

The online version contains supplementary material available at 10.1186/s13046-022-02500-4.

## Background

Bladder cancer (BC) is the most common type of urinary cancer worldwide, with high recurrence and mortality [1]. Although numerous novel treatment regimens have been applied in the recent decade, no radical improvement in prognosis has been achieved in clinical practice. To date, local or systemic chemotherapy using widely accepted drugs is still the best therapeutic intervention to assist in surgery for BC treatment [2]. Although immune checkpoint inhibitors (ICIs) have emerged as an effective alternative for managing advanced disease and have shown durable responses in a subset of patients with BC [3], unfortunately, the overall response rate is only approximately 15–25% [4]. To improve the outcome of patients with BC, we need to urgently explore more therapeutic targets and identify appropriate drugs for treating BC [5].

Unbalanced methylation is gradually being accepted as a potential driver in human cancers [6]. Although lysine methyltransferases are widely known to regulate gene expression coupled with BC development, arginine methyltransferases and their roles in BC remain obscure [7]. Protein arginine methyltransferases (PRMTs) are enzymes that transfer a methyl group from S-adenosyl-L-methionine (SAM) to the substrate arginine side chain and can be categorized into different subtypes based on their catalytic routes [8]. Arginine methylation disorder has been reported to be prevalent in breast, lung, and colon cancers and leukemia [9]. Most PRMTs have been implicated in the regulation of cancer-associated epigenetics and chromatin transcription signaling, RNA metabolism, and DNA repair [10]. Recent reports have shown that some PRMTs play indispensable roles in regulating BC and are closely related to the proliferation, invasion, and poor prognosis of the disease [11]. However, no systematic analysis on PRMTs in BC has been performed to date, and the type of PRMT that specifically promotes the development of BC remains unknown.

The roles of PRMT enzymes in numerous diseases have spurred significant interest in targeting them pharmacologically [12]. Among these enzymes, including EPZ015866, EPZ015666, GSK3326595, and LY-283, the development of PRMT5 inhibitors is the most attractive approach [13–16]. Although some inhibitors have shown an inhibitory effect on PRMT5 enzyme activity and function, their toxicity and therapeutic effect are still being investigated [17].

Kava has been used for treating inflammatory bladder diseases for more than 100 years, and chalcones are the main classes of compounds identified from kava extracts, including flavokawain A, B, and C [18, 19]. These chalcones have also been found to have strong anti-tumor activity against various cancers, such as colon, lung, gastric, and breast cancers [20–25]. FKA, in particular, has a unique anti-cancer activity against urinary tract tumors [26]. Studies have demonstrated that FKA has the strongest anti-BC activity among the kava extracts discovered till date [27].

In previous studies, FKA could induce apoptosis in BC cells via the involvement of Bax protein-dependent and mitochondria-dependent apoptotic pathways [27]. In addition, FKA could induce a G2-M arrest in the bladder and prostate cancer cells [28, 29]. In mouse models, FKA targeted the activated Ha-Ras pathway for the prevention and treatment of non-muscle invasive BC [30]. FKA is not metabolized by the liver, but is mainly concentrated in urine, indicating that orally administrated FKA could specifically act on urinary tract carcinoma [28]. Meanwhile, no organ toxicity has been observed when administering FKA [31]. Thus, FKA may be a promising anti-bladder cancer drug, and its potential anti-cancer mechanism warrants further exploration.

The present study revealed that PRMT5 was the most specific epigenetic regulator of all PRMT enzymes involved in BC and was highly correlated with the malignant characteristics of the disease. We identified FKA as a specific inhibitor targeting PRMT5, which could disrupt the binding of PRMT5 to histone H2A and H4, thereby inhibiting the symmetric arginine dimethylation level of histones. Our current findings suggest that PRMT5 is an attractive molecular target, and that FKA may be an effective small natural molecule drug for treating BC patients with abnormal PRMT5 activity.

## Methods

### Cells and reagents

Bladder tumor cell lines, UMUC3 and T24, and urothelial cell line, SV-HUC-1, were acquired from the cell bank of the Chinese Academy of Sciences and cultured in high glucose DMEM or PRMI 1640 medium with 10% FBS (Gibco, Thermo Fisher Scientific, USA). All reagents [EPZ015666 (22 nM), GSK3326595 (6.2 nM), gemcitabine (7.5 ng/mL), cisplatin (100 mg/mL)] were purchased from MedChemExpress, and various concentrations FKA were used to treat UMUC3 and T24 cells.

### Cell transfection

Transient transfection was performed using LipoFiter 3.0 (HANBIO Corporation, China) to introduce plasmid into cells. A PRMT5 expression cell line was constructed using lentivirus, and stable transfected cells were selected using puromycin.

### Patients and clinical samples

Surgically removed BC specimens and their adjacent normal counterparts were collected from the First Affiliate Hospital of China Medical University. The samples were stored at − 80 °C after collection. The study design was approved by the Institutional Ethics Committees at the First Affiliate Hospital of China Medical University. The patients’ medical records are provided in Supplementary Table; all study participants provided informed consent before specimens were collected.

### Western blotting

Cell lysates were extracted, standardized, and attenuated for electrophoresis. Proteins were separated using 10% SDS-PAGE and transferred onto PVDF membranes. The membranes were then blocked at room temperature and incubated with the primary and secondary antibodies. The antibodies used are listed in the Supplementary Table.

### Co-immunoprecipitation

Antibodies were incubated with cell lysates prepared as described above in the presence of magnetic beads (MedChemExpress Corporation, China). Proteins were separated from beads by heating with SDS-containing loading buffer.

### Immunofluorescence

Cells were fixed in 4% paraformaldehyde and permeabilized using 5% Triton X-100. Antibodies were incubated in 5% FBS overnight; DAPI staining was performed to visualize the nuclei. A confocal microscope (Olympus, Japan) of 100 magnification was used for obtaining images.

### Streptavidin agarose affinity assay

Briefly, HEK293T cells were transfected with PRMT5-Flag and treated with 40 μM Biotin–FKA for 4 h. The cells were then lysed in lysis buffer, and cell lysates were incubated with streptavidin agarose overnight at 4 °C. Next, the streptavidin agarose beads were denatured and re-suspended in SDS-containing loading buffer. The results were obtained using anti-PRMT5 or anti-Flag antibody.

### Cellular thermal shift assay (CETSA)

CETSA was conducted as previously described by Lutz et al. [32]. Plasmid EPL-tagged PRMT5-WT or EPL-tagged PRMT5-Mut was transfected into T24 and UMUC3 cell lines. The cells were diluted to 1.25 × 10^5^ cells/mL in a growth medium and inoculated into a 24-well plate, to which 40 μM FKA was added, and then incubated at 37 °C and 5% CO_2_ for 1 h. The plate was then gradually heated from 40 to 68 °C, with temperature increments of 2 °C for 3 min each time. The plate was then immediately supplemented with the cell lysis and detection reagent. After 30 min of incubation at room temperature, the chemiluminescence was determined using a Multimode Microplate Reader. Data were collected, and melt curves were plotted in GraphPad Prism using a Boltzmann sigmoidal fit.

### Bio-layer interferometry assay

An Octet K2 system was used for conducting the binding experiments on the PRMT5 proteins with FKA samples. All PRMT5 samples were prepared in Octet buffer (20 mM Tris-HCl, pH 8.0) at a concentration of 10 μM/L. Random-biotinylated FKA proteins were prepared at concentrations of 2, 3, and 4 μM. The hydrated streptavidin biosensors (ForteBio) first captured the biotinylated spike proteins for 60 s, and were then transferred to Octet buffer for 60 s to remove unbound proteins and provide the baseline. Then, they were immersed in diluted FKA samples for 120 s to provide the association signal, followed by transfer into Octet buffer to test for a disassociation signal for 240 s. The generated data were analyzed using the ForteBio Data Analysis software for correction and curve fitting.

### qRT-PCR

Total RNA was extracted using TRIzol reagent, cDNA was synthesized using the PrimeScript RT Master Mix kit (Takara Bio, China). The PCR panel was obtained using the SYBR Premix Ex Taq kit (Takara Bio, China), according to the manufacturer’s instructions. The primer sequences used are listed in the supplement table.

### CCK-8 assay

Cells were added into 96-well plates and imposed with certain conditions. CCK-8 reagent (GlpBio Tech.) was added with fresh medium after treatment, and absorbance was measured at 450 nm.

### Apoptosis assay

The apoptotic percentage of cells was assessed using an AnnexinV-FITC Apoptosis Detection Kit (BD Biosciences, USA). Briefly, treated cells were centrifugated at 1000×*g* for 5 min, washed twice with PBS, and resuspended in the 400 μL 1× binding buffer; 5 μL of Annexin V-FITC and propidium iodide were added in turn and mixed. The treated cells were placed in the dark at room temperature for 5–15 min, after which flow cytometry analysis was performed.

### Xenograft mice model

Four-week-old Balb/c nude mice were prepared at the China Medical University and experiments were conducted in accordance with the institutional guidelines approved by the Animal Care and Use Committee (IACUC Issue No. KT2022608). Approximately 5 × 10^6^ cells per mouse, wild-type or PRMT5 knockdown UMUC3 cells, were injected subcutaneously, and FKA (30 mg/kg, dissolved in DMSO) was injected around the tumor in the wild-type group every 3 days. After 24 days of continuous observation, the mice were sacrificed, and the weights of xenografted tumors and mice were recorded.

### Bioinformatics analysis

The Cancer Genome Atlas Program (TCGA) dataset was downloaded for mining in R [33]. WGCNA and ssGSEA were used for data analysis [34, 35]. A detailed description is provided in the Supplementary Materials.

### Molecular docking and dynamic simulation

The Glide docking method in Schrodinger software was used to predict the binding pattern of FKA and PRMT5 (PDB ID: 4X60), which generated a PDB file of the FKA-PRMT5 complex. Molecular dynamics simulations of the complex were performed using Amber14. The general AMBER force field (GAFF) was used for FKA, and the ff14SB force field was used for PRMT5. Molecular mechanics optimization and molecular dynamics simulation of all compounds were performed using the Sander or Pmemd procedures in Amber14. The binding free energy was calculated using the MM/PBSA method.

### Pattern drawing

The pattern diagram was drawn using the Figdraw software.

### Statistical analysis

Statistical analysis was performed using GraphPad Prism software. All data were presented as the mean ± SD of at least three independent experiments, and all differences between the indicated groups were tested using the Student *t*-test or one-way ANOVA. Statistical significance was considered at *p* < 0.05 (* represented *p* < 0.05; ** represented *p* < 0.01; and *** represented *p* < 0.001).

## Results

### PRMT5 was a BC prognostic factor among arginine methyltransferases

**Fig. 1:**
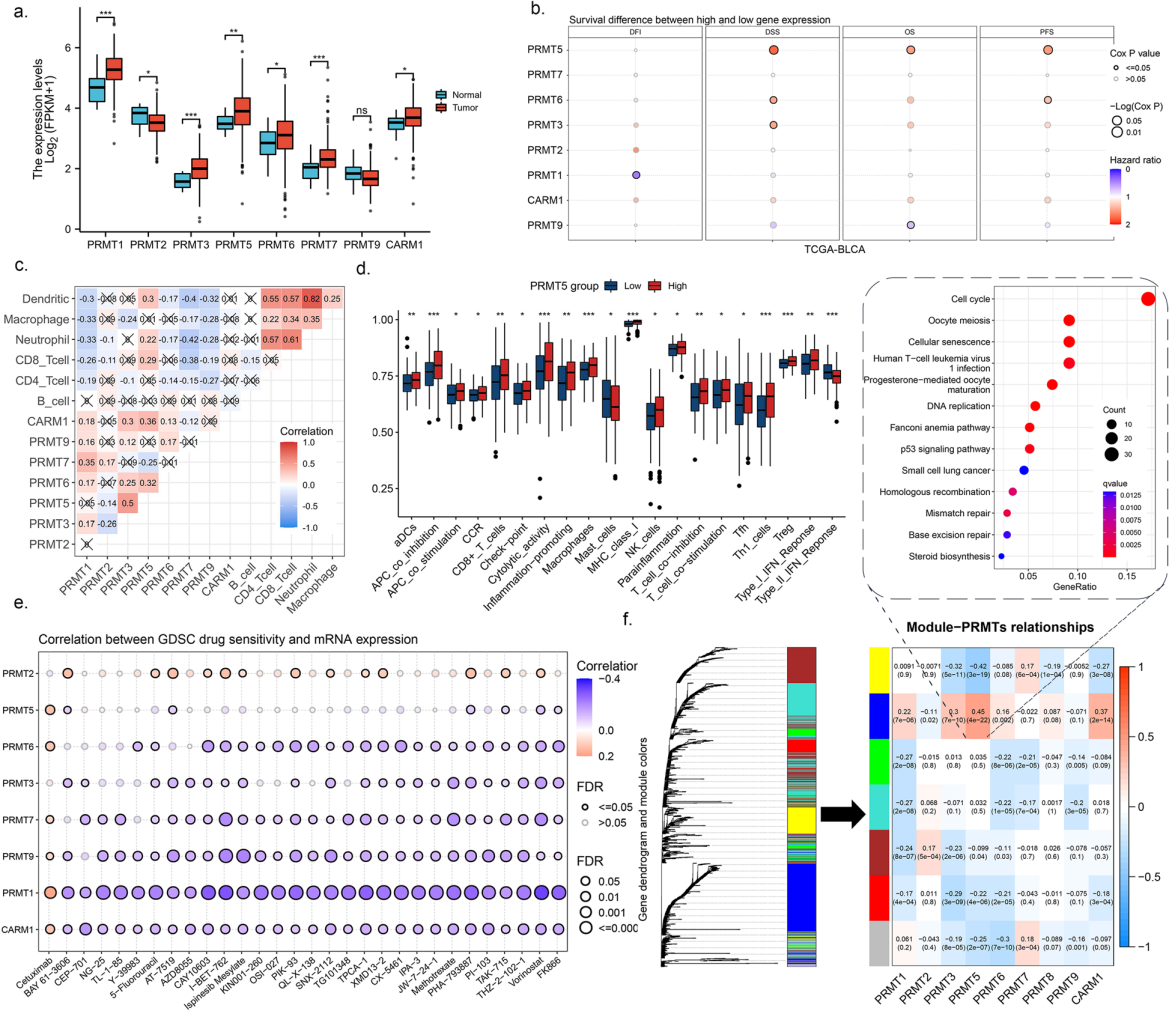
Among arginine methyltransferases, PRMT5 is a prognostic factor in BC. **a** PRMT family expression in normal bladder and BLCA from TCGA dataset. Data are presented as the mean ± SD. * *p* < 0.05, ** *p* < 0.01, *** *p* < 0.001, ns: not significant using Wilcoxon test. **b** BLCA patient’s prognosis according to PRMT family expression in TCGA. DFI: disease-free interval, DSS: disease-specific survival, OS: overall survival, PFS: progression-free survival. **c** Pearson correlation between PRMT family expression and immune cells evaluated by estimating TCGA BLCA dataset. **d** ssGSEA scored different immune cell components grouped by PRMT5 expression in TCGA BLCA dataset. Data are presented as the mean ± SD. * *p* < 0.05, ** *p* < 0.01, *** *p* < 0.001, ns: not significant using Wilcoxon test. **e** Correlation between PRMT family expression in TCGA BLCA dataset and drug sensitivity data provided in the GDSC database. **f** WGCNA clustered PRMT family gene modules, and KEGG pathway enrichment analysis performed for the most significant gene module associated with PRMT5 expression

To assess whether PRMT family expression was associated with the development of BC, we performed an analysis of the PRMT family expression, which showed that most PRMTs were upregulated in tumor tissues compared with those in normal counterparts (Fig. 1a). However, as the disease progressed, only PRMT5 was correlated with deteriorating prognosis (Fig. 1b). Furthermore, only PRMT5 was positively correlated with immune cell infiltration in BC, and immune gene set analysis showed that various types of immune cell were more active in the group with high PRMT5 levels, indicating a potential sensitive response target for immune therapy (Fig. 1c, d). Drug sensitivity screening showed that an increase in PRMTs levels leads to resistance to most drugs. Cetuximab, an EGFR inhibitor, was uniquely positively correlated with PRMT expression, which indicated that EGFR inhibitors may have a considerable effect on PRMT-positive BC patients (Fig. 1e).

This indicated that PRMT5 was a BC-prognosis associated arginine methyltransferase. Weighted gene co-expression network analysis on PRMTs identified the most correlated gene module with PRMT5 expression, and functional enrichment analysis showed that this module was correlated with the cell cycle and chromatin region, indicating the involvement of PRMT5 in regulating chromatin remodeling and alternative splicing (Fig. 1f and Supplement Figure S1a). After analyzing the chromatin remodeling results, we noticed that most BC marker genes changed in line with PRMT5 expression, and genes correlated with growth signal were upregulated (Supplement Figure S1b).

### Correlation of PRMT5 expression in BC tissues and cell lines with poor prognosis

**Fig. 2:**
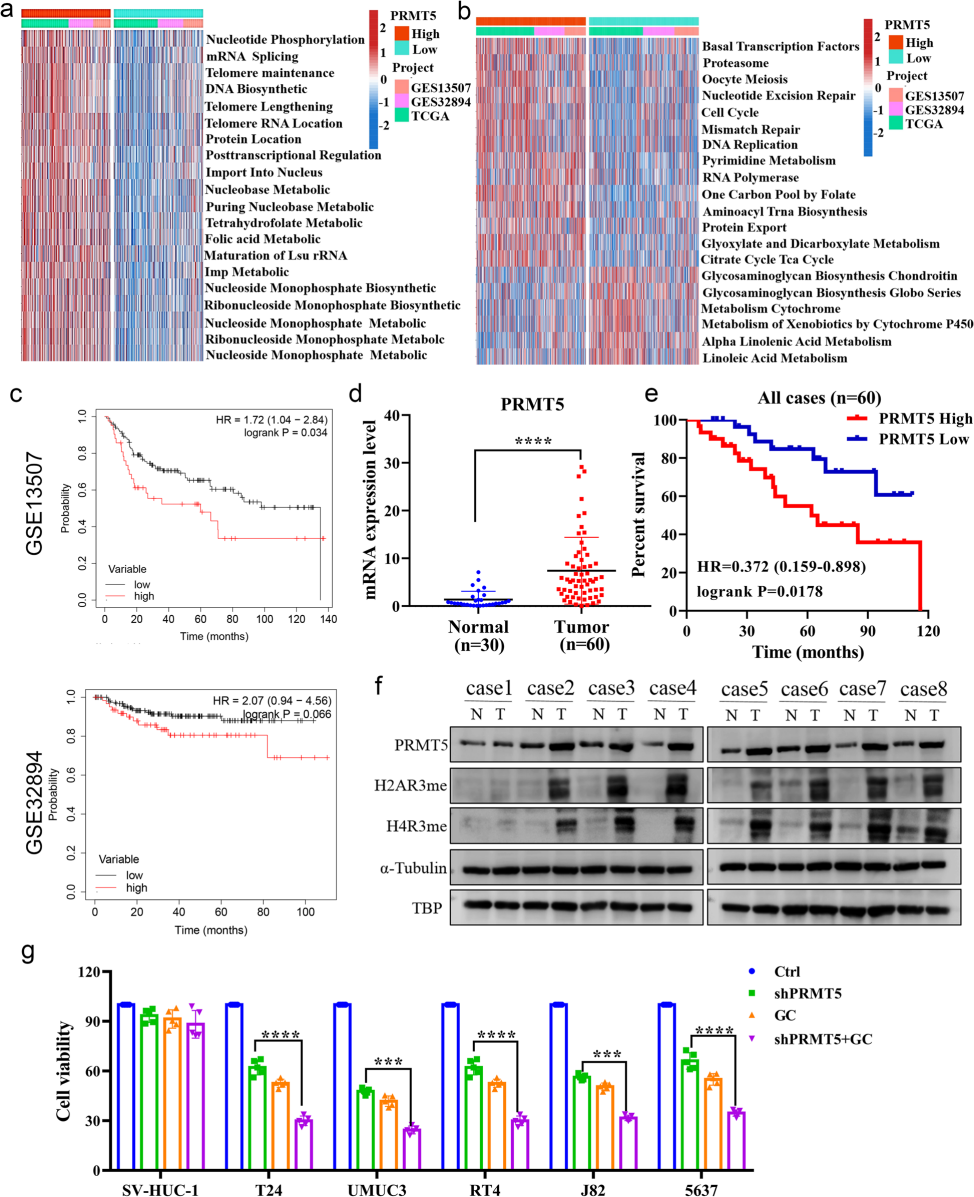
PRMT5 expression correlated with poor prognosis in BC tissues and cell lines. **a** Gene ontology analysis for the biological process of differentially expressed genes based on PRMT5 expression in three BC datasets. **b** KEGG annotation of differentially expressed genes based on PRMT5 expression in three BC datasets. **c** Kaplan–Meier analysis of survival prognosis based on PRMT5 expression in GEO datasets. **d** qPCR analysis of PRMT5 expression in BC tissues and adjacent normal bladder tissues. **e** Kaplan–Meier analysis of survival based on PRMT5 expression in patients. **f** Western blot analysis of PRMT5 expression and histone methylation level in BC tissues and adjacent normal bladder tissues. **g** Cell viability analysis of different BC cell lines by knocking down PRMT5 or treatment with gemcitabine and cisplatin

To explore the function of PRMT5, we combined several transcriptome datasets into a validation set and annotated different PRMT5 expression groups. We observed that a high PRMT5 expression level could activate DNA and RNA regulation pathways that modulate chromatin stability (Fig. 2a, b). Kaplan–Meier analysis on these data also showed poor prognosis with high PRMT5 expression levels (Fig. 2c). In immune therapy cohort, though not presenting a significant *p*-value, high PRMT5 expression shortened the median survival time by approximately 4 months, which may impede the effectiveness of atezolizumab (Supplement Figure S1c). To confirm these results, we tested the expression of PRMT5 in BC tissues. We found that PRMT5 was aberrantly overexpressed in tumor samples, resulting in poor overall survival in our cohort and that it was correlated with the progress of the disease, as shown in the stained BC tissue (Fig. 2d, e and Supplement Figure S1d). Compared with normal tissue, the methylation level of arginine on histone H4 and H2A increased in tissues expressing high levels of PRMT5; this may suggest that PRMT5 contributed to the abnormal histone arginine methylation in BC (Fig. 2f). Furthermore, PRMT5 silencing significantly attenuated cell viability in BC tissues and different cell lines, but had a minimal effect on normal urothelial cells; silenced PRMT5 in the gemcitabine and cisplatin (GC)-treated group showed a synergetic inhibitory effect (Fig. 2g).

### Evaluation of FKA as a candidate drug for targeting PRMT5

**Fig. 3:**
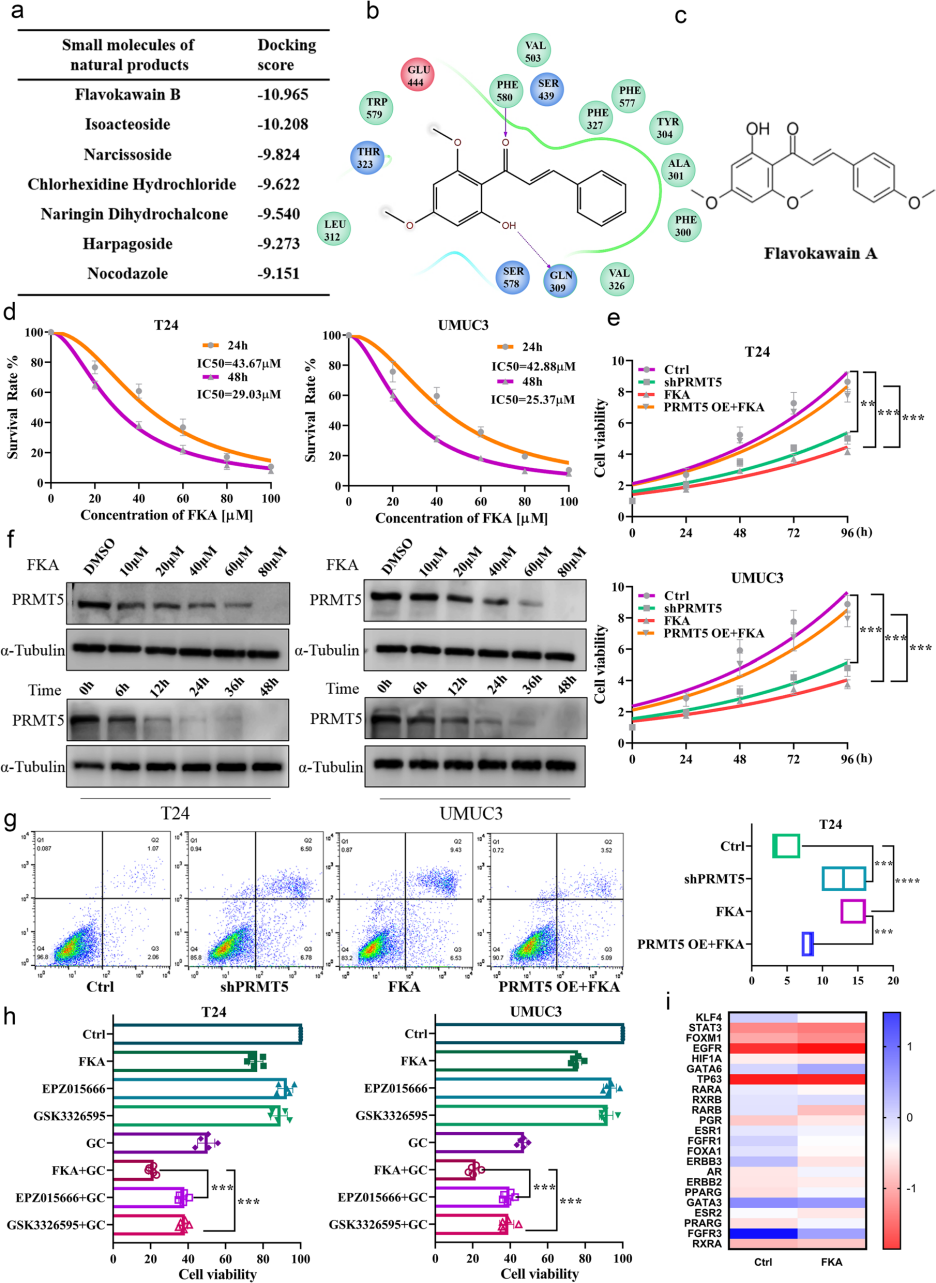
Evaluating FKA as a candidate drug for targeting PRMT5. **a** PRMT5 candidate inhibitors ranked by docking score. **b** Docking model exhibited the binding model between FKB and PRMT5. **c** Chemical structure of FKA. **d** Cell viability assay performed after treatment with FKA, and FKA IC50 were calculated in T24 (left) and UMUC3 (right). Data are presented as the mean ± SD of three replicates. **e** PRMT5 expression changes after treatment with different concentrations of FKA at different times, in T24 (left) and UMUC3 (right). **f** Cell viability measured in T24 (upper) and UMUC3 (lower) after PRMT5 expression knockdown or treatment with 40 μM FKA. FKA treatment rescue effect tested in BC cells overexpressing PRMT5. Data are presented as the mean ± SD of three replicates. ** *p* < 0.01, *** *p* < 0.001, using one-way ANOVA test. **g** Cell apoptosis measured after PRMT5 expression knockdown, 40 μM FKA treatment, and FKA treatment in T24 overexpressing PRMT5 using flow cytometry (left). Apoptosis rates for replicated assays were counted (right). Data are presented as the mean ± SD of three replicates. ** *p* < 0.01, *** *p* < 0.001, using Student’s *t*-test. **h** Cell viability changes after treatment with 40 μM FKA, two PRMT5 inhibitors and BC first-class chemotherapy drug. Synergetic effect on cell viability upon co-treatment with FKA or PRMT5 inhibitors and GC in T24 (left) and UMUC3 (right). Data are presented as the mean ± SD of three replicates. ** *p* < 0.01, *** *p* < 0.001, using one-way ANOVA test. **i** BC regulon gene changes after adding FKA to T24, measured using PCR, and the fold changes were standardized and normalized by log10. Data are presented as the mean ± SD of three replicates

To date, no drugs targeting PRMT5 in clinic trials are reported to be effective against BC [9]. We utilized high-throughput screening to identify small molecular compounds that could specifically bind to PRMT5. After ranking, we found that flavokawain B (FKB), an extract from the kava plant, presented the highest binding score (Figs. 3a, b). Zi et al. recognized FKA as a major component in kava and showed that it had a major effect on inhibiting BC tumorigenesis (Figs. 3c) [30]. We tested the effective dose of three kava extracts, FKA, FKB, and FKC, and found that BC cells were most sensitive to FKA (Figs. 3d and Supplement Figure S2b, c); FKA had no growth inhibitory effect on normal urothelial cells (Supplement Figure S2a). In addition, dose- and time-dependent PRMT5 degradation was observed in relation to FKA, indicating a clearance of inactive PRMT5 caused by FKA administration (Fig. 3e). Meanwhile, BC cells treated with FKB or FKC presented no impact on PRMT5 expression (Supplement Figure S2d, e). FKA treatment had no influence on other PRMT proteins (Supplement Figure S2f). Collectively, our observations indicated that FKA could specifically inhibit PRMT5.

PRMT5 knockdown or FKA treatment attenuated BC cell viability; however, the artificial overexpression of PRMT5 limited the effect of FKA at the same concentration (Fig. 3f). Furthermore, both PRMT5 knockdown and FKA treatment could induce apoptosis in BC cells; however, using the same FKA concentration, its effect was abated in the high PRMT5 expression group, indicating a dose-dependent relationship between the effect of FKA and PRMT5 expression (Fig. 3g and Supplement Figure S3a, b). Compared with two PRMT5 inhibitors, EPZ015666 and GSK3326595, a better response against BC cells was observed in the FKA-treated group (Fig. 3h). The synergistic effect of PRMT5 inhibitors with GC regimen indicated that FKA had a synergistic effect with GC, and that it was more efficient than the two PRMT5 inhibitors when combined with these chemotherapeutic drugs [36]. Moreover, a synergistic inhibitory effect was observed when combining PRMT5 silencing or FKA with cetuximab, the EGFR inhibitor sensitive to PRMT5 expression, as indicated by bioinformatics (Supplement Figure S3c). These data indicate the great potential for exploring combination therapy for targeting PRMT5.

### FKA binding to PRMT5

**Fig. 4:**
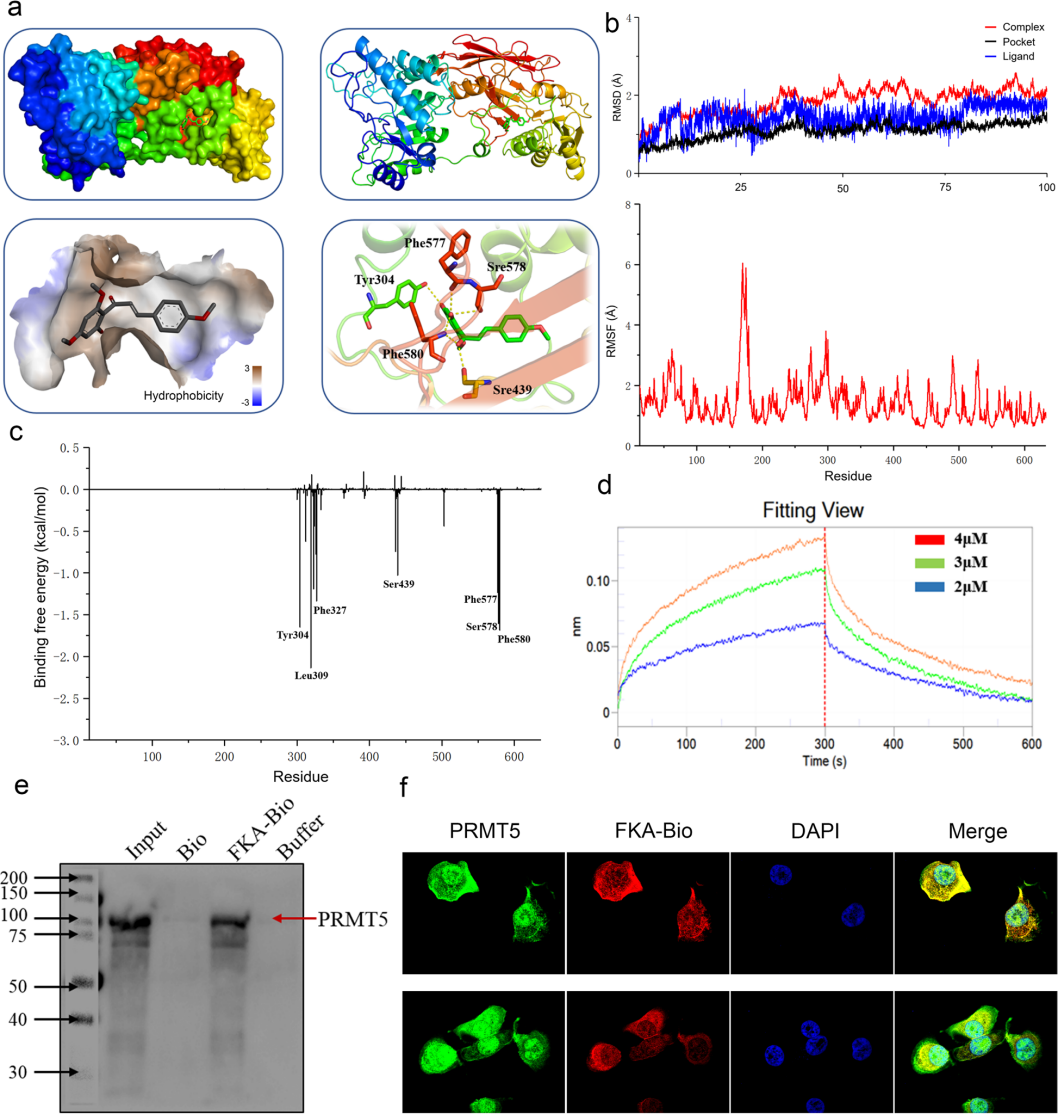
FKA binding to PRMT5 in BC cells. **a** The Glide docking method in Schrodinger software was used to predict the binding pattern between PRMT5 and FKA. The high-resolution docking model of FKA binding to PRMT5 was used to identify potential binding sites. **b** The Gaussian09 software package of Amber software was used to analyze the molecular dynamic simulations of FKA binding to PRMT5, and the corresponding RMSD (Upper) and RMSF (lower) scores were calculated. **c** Binding free energy analysis of FKA to amino acid residues of PRMT5. **d** Bio-layer interferometry detected the combination and dissociation process of FKA and human recombinant PRMT5 to determine the affinity between FKA and PRMT5. **e** Pull-down assays confirmed that biotin cross-linked FKA could precipitate with PRMT5 in 293 T cell lysis. **f** Confocal microscopy images indicate the co-localization of FKA with PRMT5 in T24 cells. PRMT5 (green fluorescence), FKA (red fluorescence), DAPI (blue fluorescence), and the merge images presents the integration of the three kinds of fluorescence

To elucidate the mechanism of the binding process, we constructed a docking model and showed that FKA was localized at the arginine-binding pocket of PRMT5 and formed five hydrogen bonds at Tyr304, Ser439, Phe577, Ser578, and Phe580 with PRMT5 (Figs. 4a). The molecular dynamic simulations showed that FKA stably binds with PRMT5 over a short time, enduring little fluctuation (Figs. 4b). Seven amino acids, including Tyr304 (− 1.65 kcal/mol), Leu309 (− 2.13 kcal/mol), Phe327 (− 1.33 kcal/mol), Ser439 (− 1.03 kcal/mol), Phe577 (− 1.23 kcal/mol), Ser578 (− 1.60 kcal/mol), and Phe580 (− 1.68 kcal/mol) played major roles in the binding of the FKA ligand with the active pocket of PRMT5 (Fig. 4c).

**Fig. 5:**
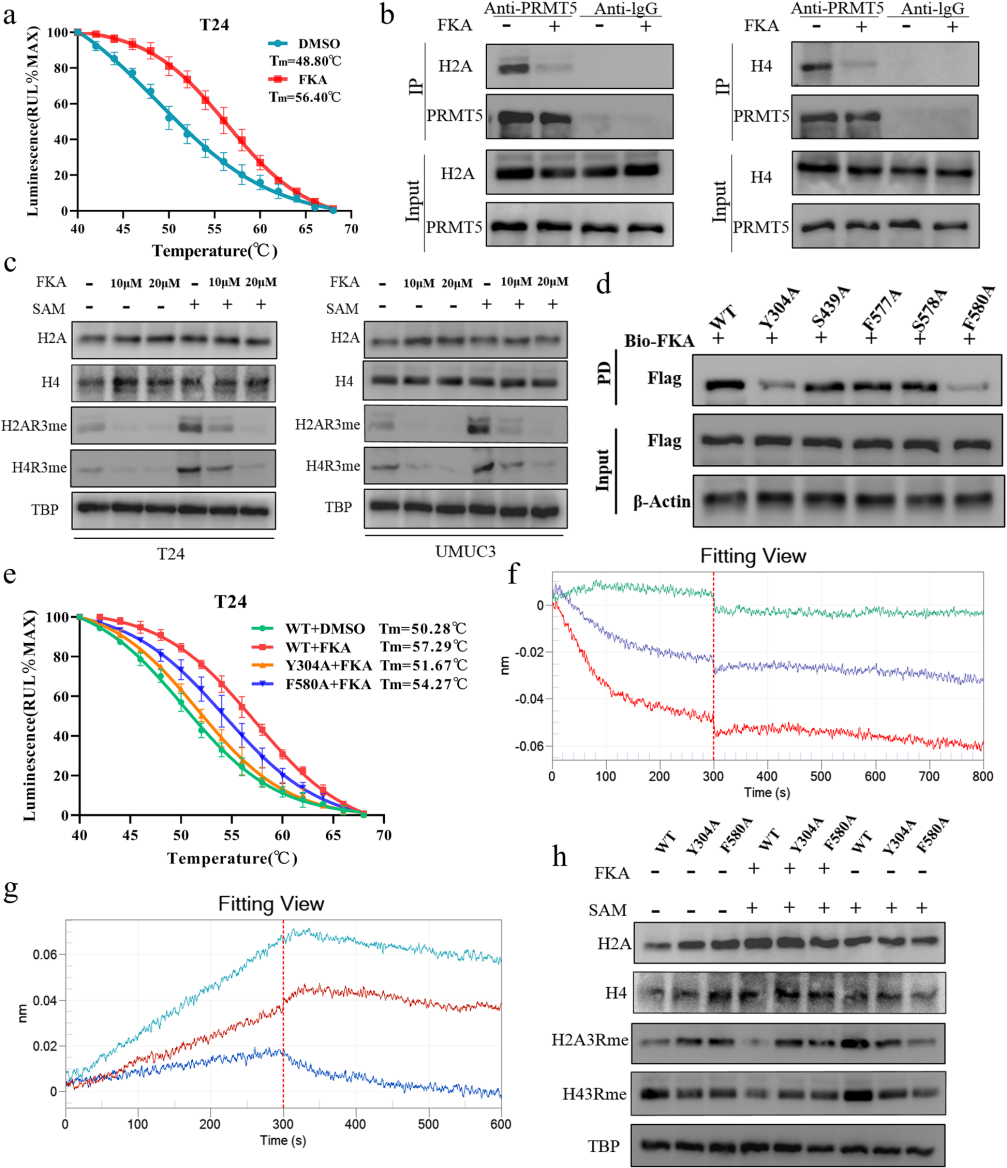
FKA inhibition of the methylation activity of PRMT5. **a** Fluorescent report CETSA assay confirmed that 25 μM FKA treatment inhibited PRMT5 degradation when heated, thus enhancing PRMT5 stability in T24. **b** Precipitation of H2A (left) and H4 (right) with PRMT5 and the inhibition of histone binding with PRMT5 after adding FKA to T24. **c** Methylation change on the third arginine of histone 2A and histone 4 when supplied with different concentrations of FKA and SAM in T24 (left) and UMUC3 (right) cells. **d** PRMT5 functional site mutant plasmids were induced in T24 cells, then a pull-down assay using biotin-labeled FKA was performed to determine the potential binding sites between FKA and PRMT5. **e** Fluorescent report CETSA assay performed for PRMT5 expression when treated with FKA in wild-type and Y304A/ F580A mutated PRMT5 in T24 cells. Data are presented as the mean ± SD of three replicates. **f** Bio-layer interferometry detected the combination and dissociation of FKA and Y304A mutated recombinant PRMT5. **g** Bio-layer interferometry detected the combination and dissociation of FKA and F580A mutated recombinant PRMT5. **h** Arginine methylation changed at the third arginine of histone 2A, and histone 4 in Y304/F580 mutated PRMT5 supplied with FKA and SAM in T24 cell

To explore the mechanism of FKA interaction with PRMT5, we performed an in vitro bio-layer interferometry assay to directly visualize the binding process between the two substances. The results showed that FKA could adhere to PRMT5 at a low dose. The affinity constant showed that the complex experienced a persistent combination and rapid reversible dissociation process, suggesting that the complex exhibited a robust combination process (Fig. 4d and Supplement Figure S4a). In cell lysis, the pull-down assay confirmed that biotin-labeled FKA could precipitate with the native PRMT5 (Figs. 4e). The CETSA assay fluorescence report also indicated that the combination of FKA and PRMT5 could promote the thermal stability of PRMT5 in BC cells (Figs. 5a and Supplement Figure S4b). Florescence images indicated the co-localization of PRMT5 and biotin-labeled FKA (Fig. 4f). All results clearly indicated that FKA directly binds with PRMT5.

### Inhibition of PRMT5 methylation activity by FKA

It is widely accepted that PRMT5 can regulate histone dimethylation. We showed that FKA-treated BC cells became less methylated on H2AR3 and H4R3, and that FKA treatment abrogated the substrates H2A and H4, which precipitated with PRMT5 (Figs. 5b, c). Furthermore, despite the elevated methylation level, after the addition of SAM, FKA still inhibited histone methylation (Fig. 5c). Methylated histones could modulate gene expression; thus, we evaluated the expression changes of 23 consensual regulon genes vitally engaged in BC when treated with FKA [37]. The results showed that most of these genes decreased, indicating a profound impact of FKA on BC cells (Fig. 3i and Supplement Figure S3e). Moreover, bioinformatics-based target prediction was used for predicting the downstream genes that FKA targeted, and pathway enrichment showed most of them enriched in key pathways related to BC (Supplement Figure S3d).

### PRMT5 mutation abolished the effect of FKA on BC cells

To further confirm the mechanism of FKA binding with PRMT5, we generated five mutated active sites in PRMT5 to elucidate the potential binding sites for FKA. By interacting with biotin-labeled FKA, we found that mutations in PRMT5, at Y304A and F580A, hindered complex formation (Fig. 5d). Changes in these two amino acid residues also led to the loss of affinity between PRMT5 and FKA (Fig. 5f, g). Moreover, the thermal stability of PRMT5 decreased in the control group compared with that in the non-mutated group treated with FKA, suggesting the reduced FKA action on mutated PRMT5 (Fig. 5e and Supplement Figure S4c). Y304 and F580 regulated PRMT5 binding to the substrate and initiated methylation; the sequence of these two regions was highly conserved in different species (Supplement Figure S4d). Our results showed that the H2A and H4 arginine-methylation were not affected when using mutant plasmids. After the addition of SAM, the mutant group showed little methylation change. Further co-treatment with FKA and SAM inhibited the histone methylation level in the wild-type PRMT5-induced group but had an interesting neutralizing effect in the mutant group (Fig. 5h).

**Fig. 6:**
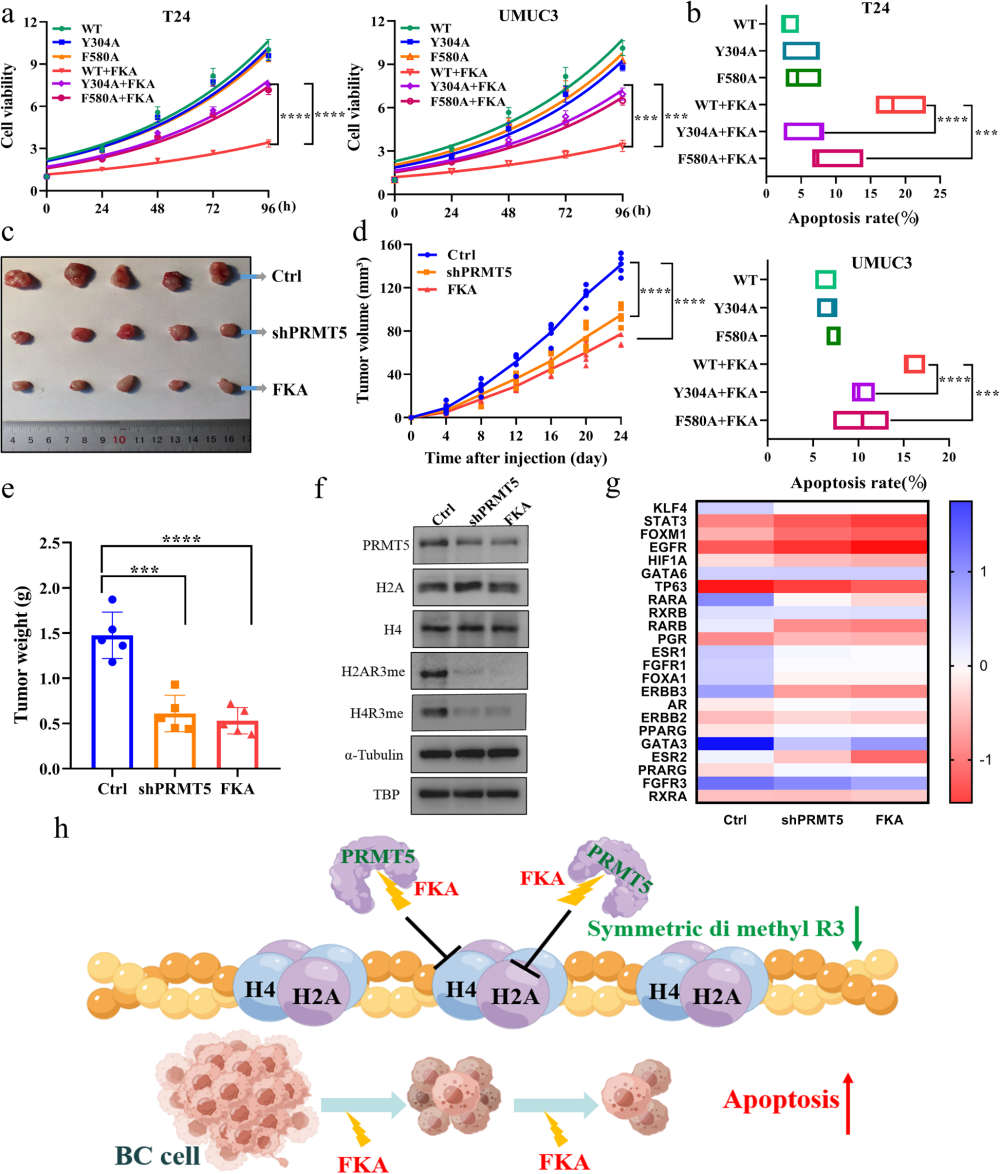
PRMT5 inhibition and FKA treatment inhibited BC growth in vivo. **a** Cell viability measured in wild-type or Y304/F580 mutated PRMT5 T24 (left) and UMUC3 (right). The inhibition effect of FKA was tested in different groups. Data are presented as the mean ± SD of three replicates. *** *p* < 0.001, **** *p* < 0.0001, using one-way ANOVA test. **b** FKA induced cell apoptosis rate in wild-type, and Y304/F580 mutated PRMT5 T24 (upper) and UMUC3 (lower) cells. Data are presented as the mean ± SD of three replicates. *** *p* < 0.001, **** *p* < 0.0001, using Student’s *t*-test. **c** Subcutaneous tumorigenesis in Balb/c nude mice injected with control UMUC3 cells or PRMT5 knockdown UMUC3 cells, and FKA was injected intraperitoneally. **d** Tumor volume was recorded during tumor growth in different groups of nude mice. Data are presented as the mean ± SD of three replicates. **** *p* < 0.0001, using one-way ANOVA test. **e** Tumor weight measured from different groups of nude mice. Data are presented as the mean ± SD of three replicates. *** *p* < 0.001, **** *p* < 0.0001, using Student’s *t*-test. **f** Arginine methylation changed at the third arginine of histone 2A and histone 4 in the subcutaneous tumor. **g** BC regulon gene changes in the control, PRMT5 knockdown, and FKA treated subcutaneous tumor tissues. **h** Graph abstract of FKA targeting PRMT5-modulated histone arginine methylation in BC

Mutated and wild-type PRMT5 had a similar effect on the cellular viability of BC; however, cells containing mutated PRMT5 became resistant to FKA treatment (Fig. 6a). We also found that mutated PRMT5 cells were less sensitive to FKA-induced apoptosis compared with the wild type (Fig. 6b). These results indicate that FKA targeting of PRMT5 in BC cells could be attenuated by mutating Y304 and F580, and the outcome may contribute to a conformational change in the mutated PRMT5 structure.

### PRMT5 inhibition and FKA control of BC growth in vivo

As we noted that FKA could inhibit BC cell lines via targeting PRMT5, we further confirmed this effect in vivo*.* We found that in subcutaneous xenografted mice, PRMT5 knockdown in BC cells or regular FKA injection could shrink tumor size compared with that in the untreated group (Fig. 6c-e). The arginine methylation level of histone and BC-associated regulon gene results revealed similar changes in both in vivo and in vitro experiments: both H2A and H4 R3 methylation decreased upon PRMT5 knockdown and in the FKA treatment groups, and most regulon genes significantly decreased (Fig. 6f, g).

## Discussion

In the present study, we thoroughly analyzed PRMT enzymes and found that their functions were inconsistent in BC. Previous studies on arginine methyltransferases in cancers have mostly focused on PRMT1, CARM1, and PRMT5 [38, 39]. In BC, however, we found that only PRMT5 was positively correlated with poor prognosis, increased drug resistance, and higher immune cell infiltration. These results highlighted the behavior of PRMT enzymes in BC and provided evidence for designing different therapeutic strategies targeting PRMT enzymes. PRMT5 regulates arginine symmetrical dimethylation on histone, a critical epigenetic regulator; thus a change in PRMT5 expression has a broad effect on downstream gene expression, in collaboration with another transcriptional regulator [40, 41]. Unlike trimethylation on lysine, which usually presents the start of transcription process, the effect of dimethylation is more flexible, including both activation and suppression functions on different histones [42]. Therefore, the aberrant expression of PRMT5 leads to different results for different types of cells and the employment of PRMT5 inhibitors may have a selective effect [43, 44].

Comparing FKA with PRMT5 inhibitors, EPZ015666 and GSK3326595, we observed that BC cells were more sensitive to FKA, and a better response was observed on combining them with the GC regimen. As a product derived from a natural source, FKA interferes with multiple pathways [29]. The multiple potential function of FKA may contribute to its higher activity against BC cells compared with that of other PRMT5 inhibitors, and the extensive effect of FKA on BC warrants further investigation. To clarify the effect of FKA, we evaluated the expression of core regulon genes in BC and found that FKA significantly inhibited their transcriptional expression, such as EGFR and FGFR3. The results indicated that FKA may have a significant effect on these BC markers. We observed a high overlap of the expression of these markers after FKA treatment and PRMT5 knockdown. Combined with other tests, we suggested that FKA could regulate these genes through the inhibition of PRMT5 activity.

Although the FKA-PRMT5 binding mode was similar to the EPZ015666-PRMT5 binding mode, the binding sites for FKA on PRMT5 were Y304 and F580, which presented strong hydrogen bond interaction [11]. Y304 had been proven to be a direct site where PRMT5 can catalyze the substrate peptide and can be phosphorylated by JAK2. Therefore, it could strongly affect the binding of PRMT5 to H2A and H4 [45, 46]. F580, a predominant binding site for EPZ015666, also affected PRMT5 binding with the substrate peptide [11]. By targeting Y304 and F580, we found that FKA affected the symmetrical arginine dimethylation levels of H2A and H4. When these two amino acids were mutated, the affinity between FKA and PRMT5 was lost, and the levels of H2AR3 and H4R3 recovered. The combination of these two binding sites enhanced FKA response in BC. Blocking these two sites uncoupled PRMT5 and histone which led to the abnormal expression of BC core genes and progression of the disease.

PRMT5 is a competitive target for developing therapeutic drugs. Three phase I/II clinical trials, NCT04676516, NCT03886831, and NCT04089449, are investigating the efficacy of different PRMT5 inhibitors in hematological and solid tumors [47, 48]. The combination of PRMT5 inhibitors with other regimens has also received great attention. NCT02783300 is evaluating PRMT5 inhibitors with immunotherapy like PD-1 antibody and NCT04794699 is testing the synthetic lethal effect of PRMT5 inhibitor with *MTAP* defect [49]. In our study, we also determined the combined effect of PRMT5 with some clinically used drugs. Inhibiting PRMT5 increased the chemotherapeutic effect of cisplatin and gemcitabine, and co-treatment of PRMT5 and EGFR inhibitor significantly inhibited cancer cell growth. The encouraging results of this work indicated that PRMT5 was a suitable target for developing combined therapies with other drugs [50]. Further exploring its synergetic effect with PARP inhibitors, immunotherapeutic antibodies, or chemotherapeutic drugs is worthy for further research.

## Conclusions

In conclusion, we systematically analyzed the role of the PRMT family in BC and confirmed that PRMT5 was highly correlated with the malignant properties of BC and could be an ideal epigenetic therapeutic target. As the first natural small molecule inhibitor of PRMT5, FKA had a strong inhibitory effect on BC and warrants further development to translate it into clinical applications.

## Supplementary Information

**Additional file 1: Supplementary Methods. Supplementary Table.** Correlation between expression of PRMT5 clinicopathological parameters in 60 cases of bladder cancer patients. **Fig. S1.** (a) PRMT5 expression correlated with BLCA gene signatures in the TCGA dataset. (b) PRMT5 expression correlated with BLCA marker genes in the TCGA dataset. (c) Kaplan–Meier analysis of survival prognosis based on PRMT5 expression in Imvigor datasets. (d) IHC analysis of PRMT5 in different stage BC. (e) Western blot analysis of PRMT5 expression and histone methylation level in BC tissues and adjacent normal bladder tissues. Statistical analysis of protein expression was shown on the right side. **Fig. S2.** (a) Cell viability assay performed by treating FKA in urothelial cell SV-HUC-1. (b) Cell viability assay performed after treatment with FKB, and FKB IC50 were calculated in T24 (left) and UMUC3 (right). Data are represented as mean ± SD in three replications. (c) Cell viability assay performed after treatment with FKC, and FKC IC50 were calculated in T24 (left) and UMUC3 (right). Data are represented as mean ± SD in three replications. (d) PRMT5 expression changed with different concentrations of FKB treatment times in T24 (upper) and UMUC3 (lower). (e) PRMT5 expression changed with different concentrations of FKC treatment times in T24 (upper) and UMUC3 (lower). (f) Different PRMT expression changed with different concentrations of FKA in T24 (left) and UMUC3 (right). **Fig. S3.** (a) Cell apoptosis measured by knocking down PRMT5 expression, FKA treatment, and supplied FKA in PRMT5 overexpressed UMUC3 using flow cytometry. (b) Apoptosis rates for replicated assays were counted. (c) Cell viability assay performed after treatment with FKA or PRMT5 shRNA combined with cetuximab in T24 (left) and UMUC3 (right). Data are represented as mean ± SD in five replications. (d) Functional pathway enrichment of predicted FKA downstream targets. (e) Bladder cancer regulon genes changes after supplying FKA in UMUC3 measured by PCR, and the fold changes were standardized and normalized by log10. **Fig. S4.** (a) Bio-Layer Interferometry detecting the combination and dissociation constants of FKA and human recombinant PRMT5. (b) Fluorescent report CETSA assay confirmed that 25 μM FKA treatment could inhibit PRMT5 degradation when heated, thus enhancing PRMT5 stability in UMUC3. (c) Fluorescent report CETSA assay performed for PRMT5 expression when treated with FKA in wild-type and Y304A/ F580A mutated PRMT5 in UMUC3 cells. (d) Conservation of PRMT5 Y304 and F580 in different species.

## Data Availability

The data in the current study are available from the corresponding author upon request.

## Abbreviations

BC: Bladder cancer
CETSA: Cellular thermal shift assay
FKA: flavokawain A
GC: Gemcitabine and cisplatin
PRMT: protein arginine methyltransferase
SAM: S-adenosyl methionine
TGCA: Cancer genome atlas program
