# Supplementary material for: Flavokawain A is a natural inhibitor of PRMT5 in bladder cancer
Source: J Exp Clin Cancer Res. 2022 Oct 5;41:293. doi: 10.1186/s13046-022-02500-4 (PMC9533510; doi:10.1186/s13046-022-02500-4)

**Supplementary Methods**

**Acquisition of gene expression data of BLCA from TCGA and GEO databases**

The RNA-seq matrix files and clinical data of bladder urothelial carcinoma (BLCA) were obtained from the cancer genome atlas (TCGA) (<https://cancergenome.nih.gov/>) which including 411 BLCA tumor samples and 19 normal samples. Two mRNA expression matrix files of BLCA, GSE13507 (n=165), GSE32894 (n=224), were extracted from gene expression omnibus (GEO) (<https://www.ncbi.nlm.nih.gov/geo/>). The “ComBat” algorithm of “sva” Package ^1^ was used to correct the batch effect.

**Pan-cancer analysis of PRMT gene family**

The web-based analysis platform gene set cancer analysis (GSCALite) ( <http://bioinfo.life.hust.edu.cn/web/GSCALite/>) ^2^ was used to perform the overall survival , survival difference between copy number variation groups and drug susceptibility analysis of PRMT gene family in multiple cancer types. Correlation analysis between PRMT gene family and tumor infiltrating immune cells was carried out by R package “corrplot”. R package “ggpubr” was used to perform the correlation analysis between PRMT gene family and clinical information.

**Single-gene analysis of PRMT5 in BLCA**

Gene set variation analysis (GSVA) is a nonparametric unsupervised analysis method mainly used to evaluate the gene set enrichment results. The “GSVA” and “GSEABase” R package were used to calculate GSVA enrichment scores of immune-related gene sets and malignancy-related gene sets in BLCA ^3^. The correlation analysis between PRMT5 and GSVA enrichment scores of malignancy-related gene sets was carried out using the R package “ggplot2”, “ggpubr” and “ggExtra”. Differential analysis of GSVA enrichment scores of GO and KEGG gene sets was performed using “LIMMA” R package. The screening condition was FDR < 0.05. The selection of differential genes between BLCA tumor samples and normal samples was carried out using “LIMMA” R package, the screening condition was fold change >2 ( | log2 fold-change | >1) and p < 0.05. Weighted gene co-expression network analysis (WGCNA) outperforms several other methods in construction co-expression network ^4^. The co-expression network of differential genes was constructed using R package “WGCNA”. The R package "clusterProfiler ^5^", "org.Hs.eg.db", "enrichplot" and "ggplot2" were used to carry out functional and pathway analyses of key module related to PRMT5.

**Statistical analysis**

In the bioinformatics part, statistical analysis was performed with R software. The correlation coefficients between two variables were calculated by the method of spearman correlation. Wilcoxon test was used to carry out the group comparisons of two groups. P<0.05 was considered as statistical significance in this study.

**References**

1. Leek JT, Johnson WE, Parker HS, Jaffe AE, Storey JD. The sva package for removing batch effects and other unwanted variation in high-throughput experiments. *Bioinformatics* 2012; **28**(6): 882-3.

2. Liu CJ, Hu FF, Xia MX, Han L, Zhang Q, Guo AY. GSCALite: a web server for gene set cancer analysis. *Bioinformatics* 2018; **34**(21): 3771-2.

3. Hanzelmann S, Castelo R, Guinney J. GSVA: gene set variation analysis for microarray and RNA-seq data. *BMC Bioinformatics* 2013; **14**: 7.

4. Allen JD, Xie Y, Chen M, Girard L, Xiao G. Comparing statistical methods for constructing large scale gene networks. *PLoS One* 2012; **7**(1): e29348.

5. Yu G, Wang LG, Han Y, He QY. clusterProfiler: an R package for comparing biological themes among gene clusters. *OMICS* 2012; **16**(5): 284-7.

**Supplementary Table. Correlation between expression of PRMT5 clinicopathological parameters in 60 cases of bladder cancer patients**

| Positive |  | PRMT5 |  |
| --- | --- | --- | --- |
| Variables | Group | LOW HIGH | *p* values |
| Gender |  |  |  |
|  | Male | 22 25 | 0.5321 |
|  | Female | 8 5 |  |
| Age |  |  |  |
|  | ≤60 | 7 11 | 0.3985 |
|  | >60 | 23 19 |  |
| T classification |  |  |  |
|  | T1/T2 | 21 9 | 0.0041** |
|  | T3/T4 | 9 21 |  |
| Distant metastasis |  |  |  |
|  | Negative | 30 25 | 0.0522 |
|  | Positive | 0 5 |  |
| Lymphatic invasion | Negative | 27 20 | 0.0575 |
|  | Positive | 3 10 |  |

| **Key source table** |  |  |
| --- | --- | --- |
| REAGENT or RESOURCE | SOURCE | IDENTIFIER |
| Antibodies |  |  |
| Anti-α-Tubulin | MilliporeSigma | Cat # T9026; RRID: AB_477593 |
| Anti-dimethyl-Histone H4 (Arg3) | Abcam | Cat # 07-213; RRID: AB_310433 |
| Anti-FLAG(R) M2 | MilliporeSigma | Cat # F1804; RRID: AB_262044 |
| Anti-Histone H2A | Abcam | Cat # ab18255; RRID: AB_470265 |
| Anti-Histone H2A (symmetric di methyl R3) | Abcam | Cat # ab22397; RRID: AB_880431 |
| Anti-Histone H4 | Abcam | Cat # ab10158; RRID: AB_296888 |
| Anti-PRMT5 | MilliporeSigma | Cat # 07-405; RRID: AB_310589 |
| Anti-TATA binding protein TBP | Abcam | Cat # ab818; RRID: AB_306337 |
| Anti-SBP-tag Antibody | MilliporeSigma | Cat # MAB10764; RRID: AB_10631872 |
| Chemicals, peptides, and recombinant proteins |  |  |
| Flavokawain A | GlpBio Technology | Cat # GC60845 |
| Gemcitabine | GlpBio Technology | Cat # GC14447 |
| Cisplatin | GlpBio Technology | Cat # GC11908 |
| EPZ015666 | GlpBio Technology | Cat # GC15302 |
| GSK3326595 | GlpBio Technology | Cat # GC32693 |
| Recombinant Human PRMT5 protein (Tagged) | Abcam | Cat # ab268889 |
| Biotin-FKA | Biosyntech, China | N/A |
| Critical commercial assays |  |  |
| FITC Annexin V Apoptosis Detection Kit I | BD biosciences | Cat # 556547 |
| Cell Counting Kit-8 (CCK8) | GlpBio Technology | Cat # GK10001 |
| PrimeScript™ RT Master Mix (Perfect Real Time) | Takara Biomedical Technology | Cat # RR036A |
| TB Green® Premix Ex Taq™ II (Tli RNaseH Plus) | Takara Biomedical Technology | Cat # RR820A |
| β- Galactosidase reporter gene detection kit | Beyotime Biotechnology | Cat # RG0036 |
| Experimental models: cell lines |  |  |
| UMUC3 | Cell Bank of the Chinese Academy of Sciences | Cat # TCHu217 |
| T24 | Cell Bank of the Chinese Academy of Sciences | Cat # SCSP-536 |
| 293T | Cell Bank of the Chinese Academy of Sciences | Cat # SCSP-502 |
| Experimental models: organisms/strains |  |  |
| Balb/c mouse | Beijing Vital River Laboratory Animal Technology | N/A |
| Oligonucleotides |  |  |
| AR Primer pair | PrimerBank | PrimerBank ID: 349501065c3 |
| EGFR Primer pair | PrimerBank | PrimerBank ID: 41327735c3 |
| ERBB2 Primer pair | PrimerBank | PrimerBank ID: 54792097c3 |
| ERBB3 Primer pair | PrimerBank | PrimerBank ID: 29351637a1 |
| ESR1 Primer pair | PrimerBank | PrimerBank ID: 170295801c3 |
| ESR2 Primer pair | PrimerBank | PrimerBank ID: 333609292c2 |
| FGFR1 Primer pair | PrimerBank | PrimerBank ID: 105990516c1 |
| FGFR3 Primer pair | PrimerBank | PrimerBank ID: 254028235c3 |
| FOXA1 Primer pair | PrimerBank | PrimerBank ID: 24497500c1 |
| FOXM1 Primer pair | PrimerBank | PrimerBank ID: 340545541c1 |
| GATA3 Primer pair | PrimerBank | PrimerBank ID: 50541958c1 |
| GATA6 Primer pair | PrimerBank | PrimerBank ID: 40288196c1 |
| HIF1A Primer pair | PrimerBank | PrimerBank ID: 194473734c2 |
| KLF4 Primer pair | PrimerBank | PrimerBank ID: 194248076c2 |
| PGR Primer pair | PrimerBank | PrimerBank ID: 160358783c2 |
| PPARG Primer pair | PrimerBank | PrimerBank ID: 116284372c1 |
| RARA Primer pair | PrimerBank | PrimerBank ID: 300388161c2 |
| RARB Primer pair | PrimerBank | PrimerBank ID: 196049371c2 |
| RARG Primer pair | PrimerBank | PrimerBank ID: 344030239c3 |
| RXRA Primer pair | PrimerBank | PrimerBank ID: 207028087c1 |
| RXRB Primer pair | PrimerBank | PrimerBank ID: 27436942c1 |
| STAT3 Primer pair | PrimerBank | PrimerBank ID: 47080104c2 |
| TP63 Primer pair | PrimerBank | PrimerBank ID: 169234656c3 |
| ACTB Primer pair | PrimerBank | PrimerBank ID: 4501885a1 |
| Recombinant DNA |  |  |
| shPRMT5 | Genechem | N/A |
| pLVX-PRMT5 WT-flag | Genechem | N/A |
| pLVX-PRMT5 Y304A-flag | Genechem | N/A |
| pLVX-PRMT5 S439A-flag | Genechem | N/A |
| pLVX-PRMT5 F577A-flag | Genechem | N/A |
| pLVX-PRMT5 S578A-flag | Genechem | N/A |
| pLVX-PRMT5 F580A-flag | Genechem | N/A |
| pLVX-ePL-PRMT5-WT | Genechem | N/A |
| pLVX-ePL-PRMT5-Y304A | Genechem | N/A |
| pLVX-ePL-PRMT5-F580A | Genechem | N/A |
| Software and algorithms |  |  |
| Graphpad Prism | GraphPad 7.0 | https://www.graphpad.com/scientific-software/prism/ |
| R (v.4.1.3) | Open source | https://www.R-project.org |
| Schrodinger | Schrodinge v2018 | https://www.schrodinger.com |
| FlowJo | FlowJo 10.0 | https://www.flowjo.com/ |
| Python | Python 3 | https://www.python.org/ |

**Supplement Figure Legends**

Figure s1. (a) PRMT5 expression correlated with BLCA gene signatures in the TCGA dataset. (b) PRMT5 expression correlated with BLCA marker genes in the TCGA dataset. (c) Kaplan–Meier analysis of survival prognosis based on PRMT5 expression in Imvigor datasets. (d) IHC analysis of PRMT5 in different stage BC. (e) Western blot analysis of PRMT5 expression and histone methylation level in BC tissues and adjacent normal bladder tissues. Statistical analysis of protein expression was shown on the right side.

Figure s2. (a) Cell viability assay performed by treating FKA in urothelial cell SV-HUC-1. (b) Cell viability assay performed after treatment with FKB, and FKB IC50 were calculated in T24 (left) and UMUC3 (right). Data are represented as mean ± SD in three replications. (c) Cell viability assay performed after treatment with FKC, and FKC IC50 were calculated in T24 (left) and UMUC3 (right). Data are represented as mean ± SD in three replications. (d) PRMT5 expression changed with different concentrations of FKB treatment times in T24 (upper) and UMUC3 (lower). (e) PRMT5 expression changed with different concentrations of FKC treatment times in T24 (upper) and UMUC3 (lower). (f) Different PRMT expression changed with different concentrations of FKA in T24 (left) and UMUC3 (right).

Figure s3. (a) Cell apoptosis measured by knocking down PRMT5 expression, FKA treatment, and supplied FKA in PRMT5 overexpressed UMUC3 using flow cytometry. (b) Apoptosis rates for replicated assays were counted. (c) Cell viability assay performed after treatment with FKA or PRMT5 shRNA combined with cetuximab in T24 (left) and UMUC3 (right). Data are represented as mean ± SD in five replications. (d) Functional pathway enrichment of predicted FKA downstream targets. (e) Bladder cancer regulon genes changes after supplying FKA in UMUC3 measured by PCR, and the fold changes were standardized and normalized by log10.

Figure s4. (a) Bio-Layer Interferometry detecting the combination and dissociation constants of FKA and human recombinant PRMT5. (b) Fluorescent report CETSA assay confirmed that 25 μM FKA treatment could inhibit PRMT5 degradation when heated, thus enhancing PRMT5 stability in UMUC3. (c) Fluorescent report CETSA assay performed for PRMT5 expression when treated with FKA in wild-type and Y304A/ F580A mutated PRMT5 in UMUC3 cells. (d) Conservation of PRMT5 Y304 and F580 in different species.

**Supplement Figures**


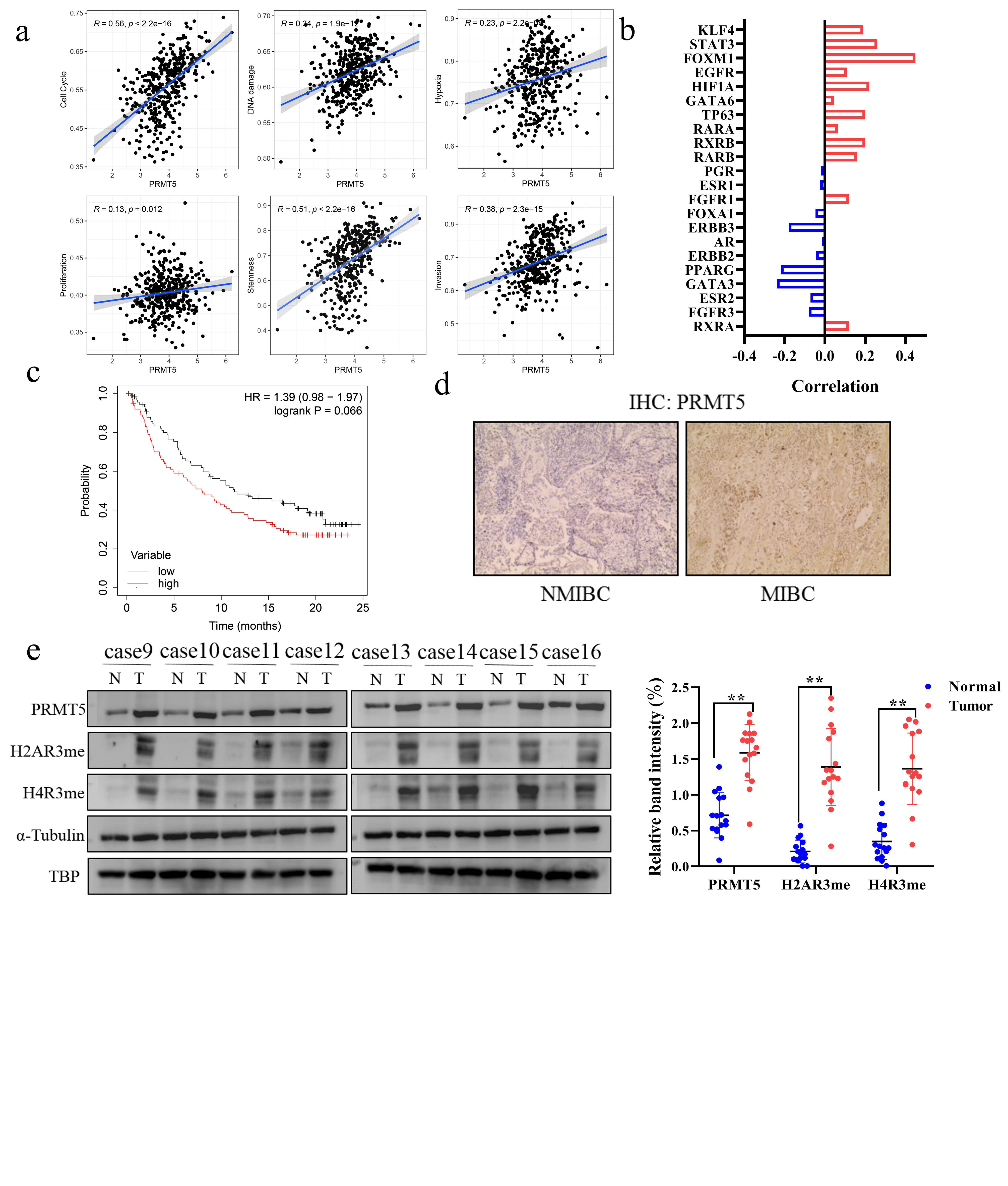


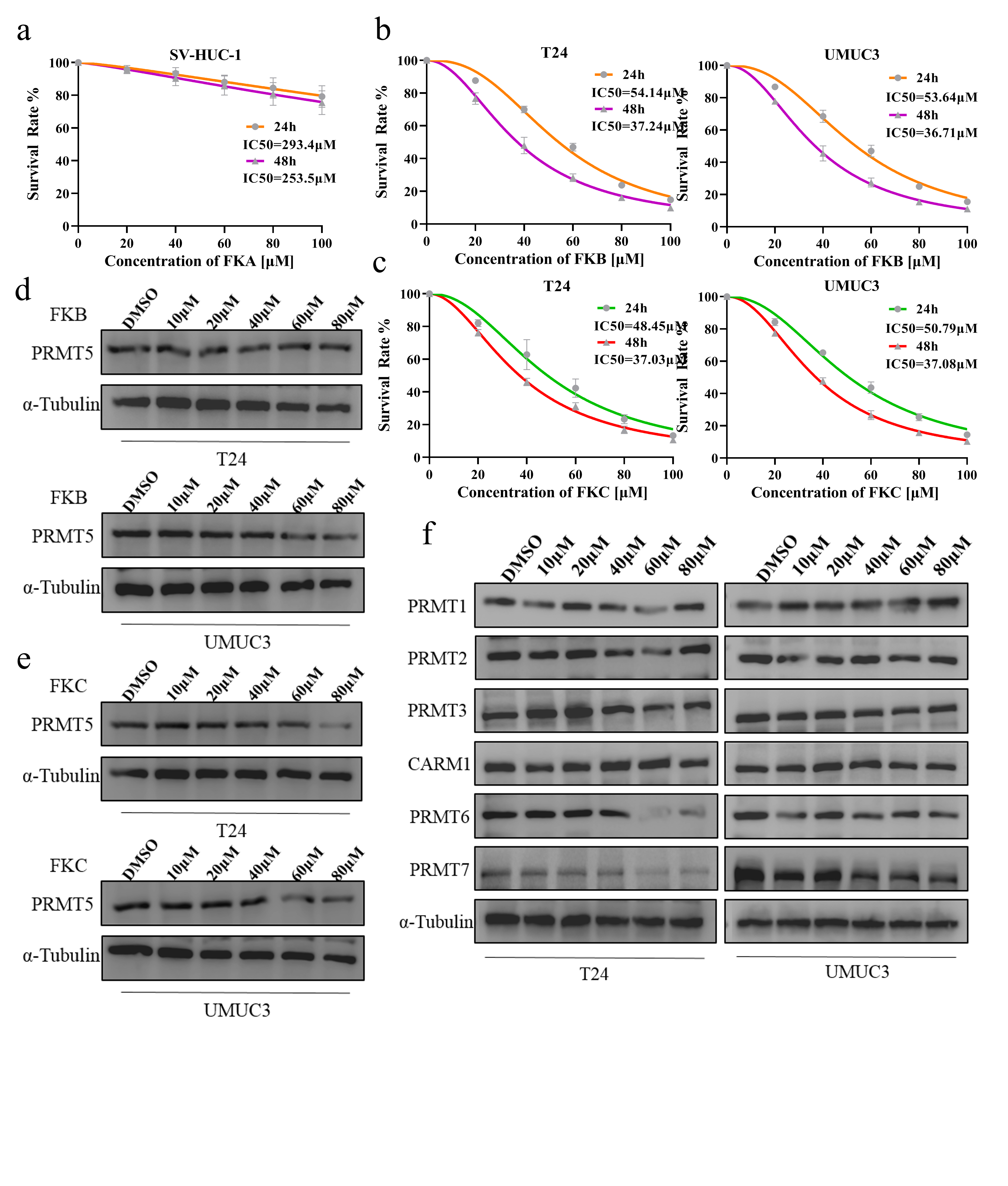


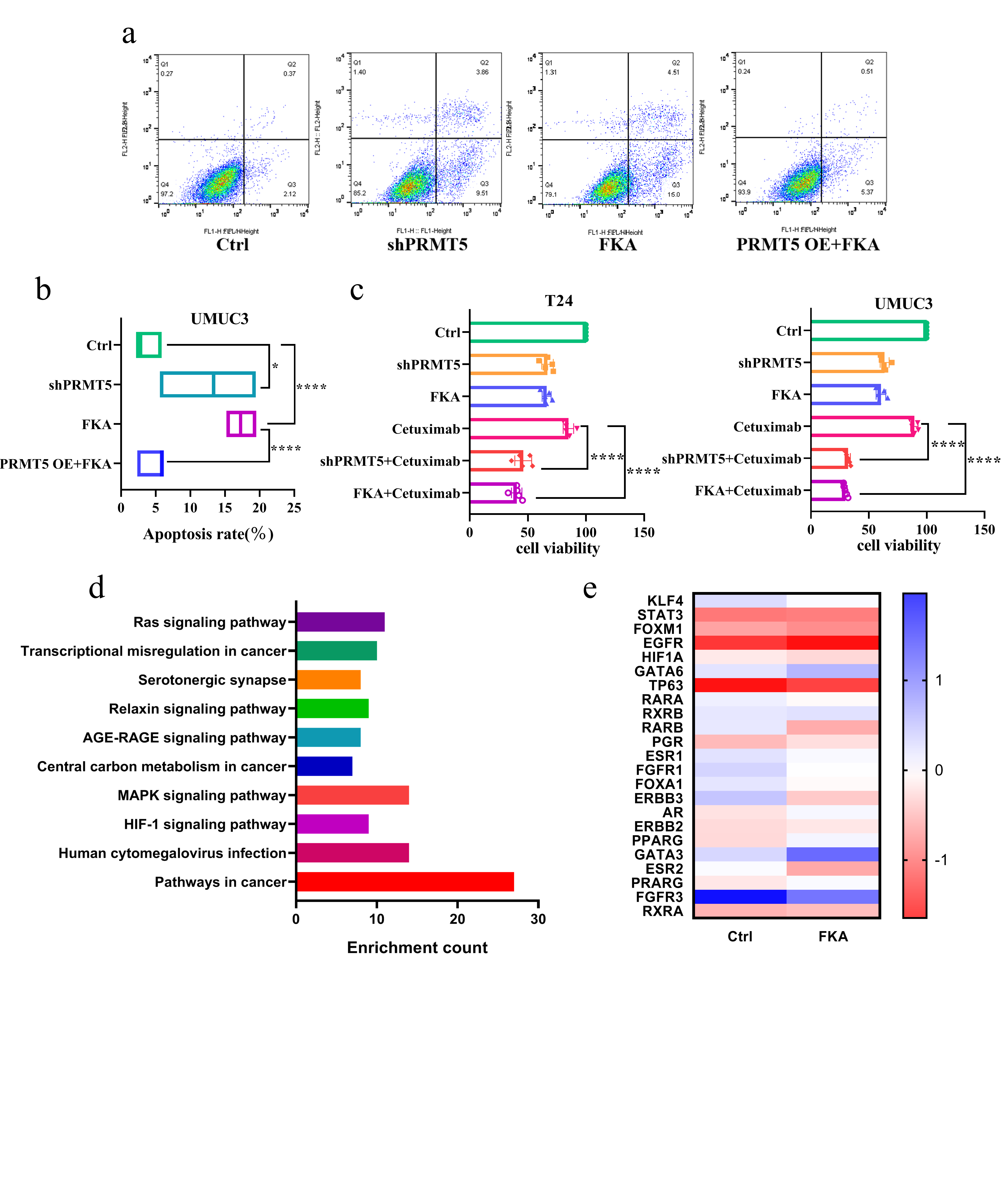


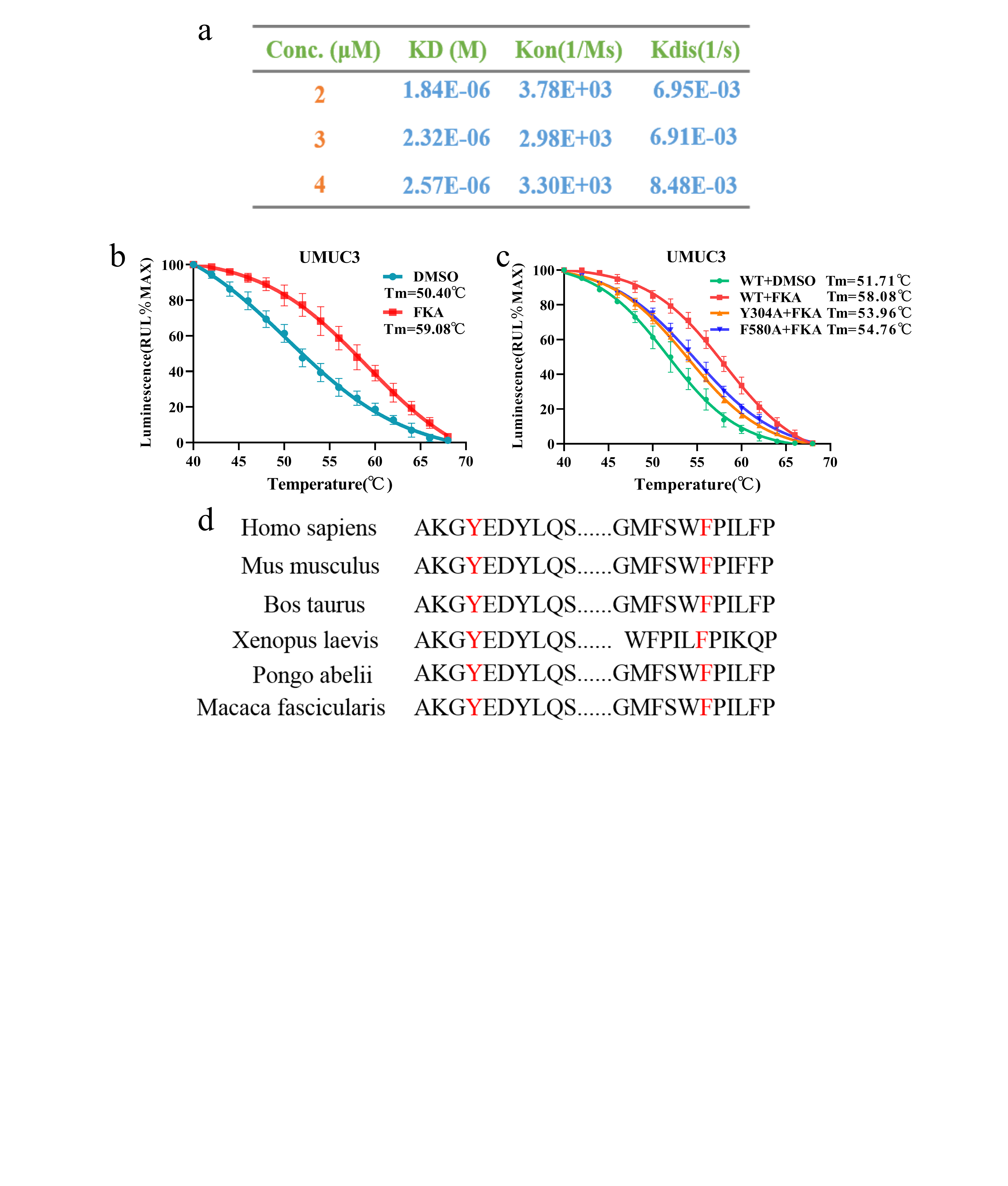

Supplement: Supplementary file 1 — Additional file 1: Supplementary Methods. Supplementary Table. Correlation between expression of PRMT5 clinicopathological parameters in 60 cases of bladder cancer patients. Fig. S1. (a) PRMT5 expression correlated with BLCA gene signatures in the TCGA dataset. (b) PRMT5 expression correlated with BLCA marker genes in the TCGA dataset. (c) Kaplan–Meier analysis of survival prognosis based on PRMT5 expression in Imvigor datasets. (d) IHC analysis of PRMT5 in different stage BC. (e) Western blot analysis of PRMT5 expression and histone methylation level in BC tissues and adjacent normal bladder tissues. Statistical analysis of protein expression was shown on the right side. Fig. S2. (a) Cell viability assay performed by treating FKA in urothelial cell SV-HUC-1. (b) Cell viability assay performed after treatment with FKB, and FKB IC50 were calculated in T24 (left) and UMUC3 (right). Data are represented as mean ± SD in three replications. (c) Cell viability assay performed after treatment with FKC, and FKC IC50 were calculated in T24 (left) and UMUC3 (right). Data are represented as mean ± SD in three replications. (d) PRMT5 expression changed with different concentrations of FKB treatment times in T24 (upper) and UMUC3 (lower). (e) PRMT5 expression changed with different concentrations of FKC treatment times in T24 (upper) and UMUC3 (lower). (f) Different PRMT expression changed with different concentrations of FKA in T24 (left) and UMUC3 (right). Fig. S3. (a) Cell apoptosis measured by knocking down PRMT5 expression, FKA treatment, and supplied FKA in PRMT5 overexpressed UMUC3 using flow cytometry. (b) Apoptosis rates for replicated assays were counted. (c) Cell viability assay performed after treatment with FKA or PRMT5 shRNA combined with cetuximab in T24 (left) and UMUC3 (right). Data are represented as mean ± SD in five replications. (d) Functional pathway enrichment of predicted FKA downstream targets. (e) Bladder cancer regulon genes changes after supplying FKA [file 13046_2022_2500_MOESM1_ESM.docx]
